# A Novel Lipoprotein Lipase Mutation in an Infant With Glycogen Storage Disease Type-Ib and Severe Hypertriglyceridemia

**DOI:** 10.3389/fped.2021.671536

**Published:** 2021-08-17

**Authors:** Fengyu Wang, Fengli Wang, Xiaojun Zhou, Yingjie Yi, Jie Zhao

**Affiliations:** ^1^Department of Pediatrics, Zibo Central Hospital, Shandong First Medical University, Zibo, China; ^2^Department of Radiology, Zibo Central Hospital, Shandong First Medical University, Zibo, China

**Keywords:** glycogen storage disease, hypertriglyceridemia, lipoprotein lipase, compound heterozygote, glucose 6-phosphate transport

## Abstract

Glycogen storage disease (GSD) Ib is a rare genetic metabolic disorder caused by gene mutation in the glucose 6-phosphate transport gene SLC37A4 (OMIM# 602671). This study aimed to explore the association between a novel lipoprotein lipase (*LPL*) mutation and severe hypertriglyceridemia in a GSD Ib infant with severe hypertriglyceridemia. A 5-month-old girl was admitted to our hospital because of repeated episodes of low-grade fever over the past month and because of neutropenia. The patient was diagnosed with GSD Ib and severe hypertriglyceridemia based on clinical manifestations and laboratory test results. Next-generation sequencing and Sanger sequencing were then applied to DNA from the peripheral blood of the patient and her parents to analyze gene mutations. Pathogenicity prediction analysis was performed using Sorting Intolerant From Tolerant (SIFT) and PolyPhen-2 platforms. The results revealed that this infant carried a compound heterozygous variation in the *SLC37A4* gene, a c.1043T > C (p.L348P) mutation derived from her mother and a c.572C > T (p.P191L) mutation derived from her father. In addition, a novel c.483delA (p. A162Pfs^*^10) frameshift mutation was found in the patient's *LPL* gene exon 4, which was derived from the heterozygous carrier of her father. The SIFT and PolyPhen-2 prediction programs indicated that these mutations were likely harmful. Medium-chain triglyceride milk and granulocyte colony-stimulating factor subcutaneous injection alleviated the symptoms. Our findings identified a novel *LPL* gene frameshift mutation combined with *SLC37A4* gene compound heterozygous mutations in a GSD Ib infant with severe hypertriglyceridemia.

## Introduction

Glycogen storage disease (GSD) I is a rare congenital metabolic abnormality caused by deficiencies in the glucose 6-phosphatase enzyme (resulting in GSD Ia) or in proteins for glucose 6-phosphate microsomal transport (G6PT) (1). GSD Ib is caused by a gene mutation in the G6PT gene *SLC37A4* (OMIM# 602671), which manifests as excessive glycogen and fat accumulation in the liver, kidney, and intestinal mucosa and as neutropenia, neutrophil dysfunction, and other symptoms (2). In addition, deleterious mutations in the same G6PT gene *SLC37A4* have also been observed in clinical cases of GSD Ic (OMIM# 232240), which were identified to have a deficiency in the P(i) transporter (3). The analysis of the *SLC37A4* gene may provide an important clinical strategy for patients with GSD Ib (4).

Clinically, patients are diagnosed with hypertriglyceridemia based on the upregulated fasting plasma triglyceride (TG) level; TG levels exceeding 885 mg/dl are categorized as severe hypertriglyceridemia (5). Recently, the role of molecular genetic determinants of severe hypertriglyceridemia has been extensively recognized (6). As a rate-limiting enzyme of TG degradation, lipoprotein lipase (LPL) plays a critical role in lipid metabolism. At the genetic level, severely increased TG levels resulting from type I hyperlipoproteinemia was shown to be caused by LPL deficiency (OMIM#238600) (7, 8). Though *LPL* mutation in patients with hypertriglyceridemia has been reported (9), a combination of an *LPL* gene frameshift mutation with compound heterozygous mutations of the *SLC37A4* gene is rare.

Medium-chain TG (MCT) milk has been proven to significantly decrease serum TG levels and has been applied as treatment in children with profound hypertriglyceridemia (10). Nevertheless, the efficacy of MCT milk for patients with GSD Ib has always been controversial (11). In this article, we reported a Chinese infant with GSD Ib who presented with severe hypertriglyceridemia with a novel frameshift mutation found in her *LPL* gene. When the patient was treated with MCT milk, her TG levels dramatically decreased.

## Case Report

A 5-month-old girl was admitted to our hospital because of repeated episodes of low-grade fever over the past month and because of neutropenia. She was a full-term infant of a gravida 1 para 1 mother, and her parents were not closely related. The patient had a history of hypoglycemia, hyperlactic acidosis, and sepsis in the neonatal period.

The physical examination showed that she was a well-thriving child but had hepatomegaly. Laboratory tests showed normal fasting blood sugar, an absolute neutrophil count of 400/mm^3^, significant hyperlipidemia (serum cholesterol level, 266 mg/dl; TG level, 2,526 mg/dl), and elevated lactate levels (3.44 mmol/L). Transaminase levels, including aspartate aminotransferase (175 IU/L) and alanine aminotransferase (53 IU/L), were both slightly elevated. The abdominal ultrasound revealed an enlarged liver (subcostal 5.1 cm; subcostal length was measured above the midline of the right clavicle, Supplementary Figure 1) and kidneys (left, 7.3 cm × 3.1 cm; right, 7 cm × 2.6 cm). The patient was diagnosed with GSD Ib and lipid metabolism disease according to the manifestations and laboratory test results.

**Figure 1:**
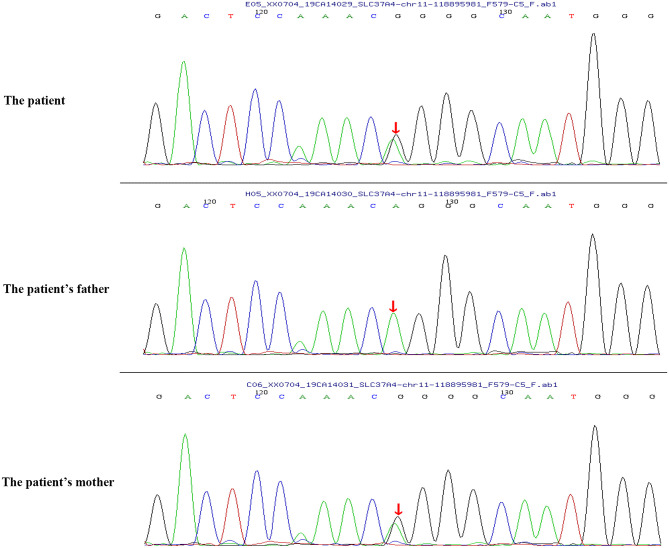
Heterozygous mutation c.1043T>C (p.L348P) in the *SLC37A4* gene of the patient and her mother.

**Figure 2:**
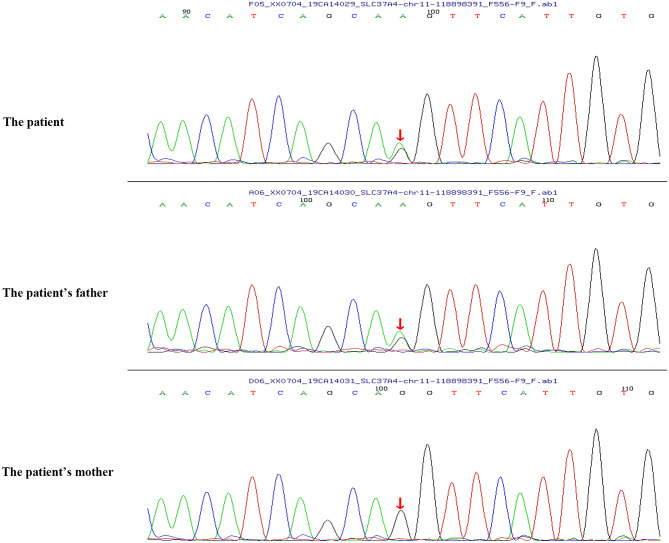
Heterozygous mutation c.572C>T (p.P191L) in the *SLC37A4* gene of the patient and her father.

**Figure 3:**
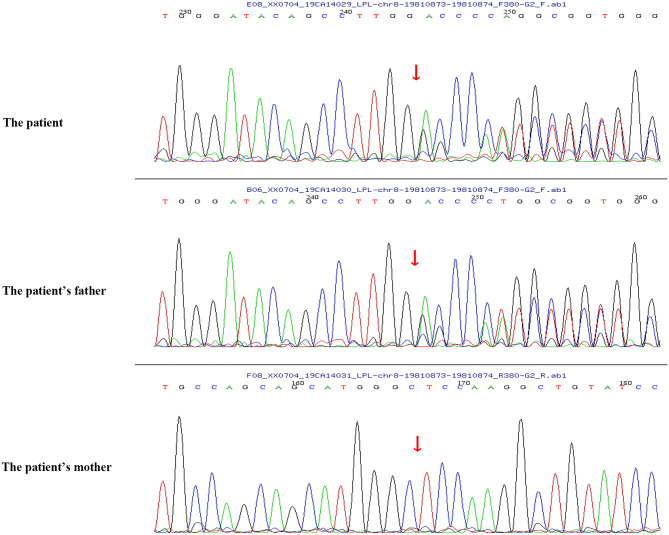
A novel c.483delA (p.A162Pfs*10) frameshift mutation in exon 4 of the LPL gene of the patient and her father.

Peripheral blood samples (2 ml per person) were obtained from the patient and her parents. Written informed consent to participate in this study was attained from the participants' legal guardian or next of kin, and this study was approved by the Medical Ethics Committee of Zibo Central Hospital, Shandong First Medical University. The TIANamp Blood DNA Extraction Kit (TIANGEN Biotech Co., Ltd., Beijing, China) was utilized to extract genomic DNA. Genomic libraries were constructed, and enriched targeted genes were sequenced using next-generation sequencing. All of the mutations that might have caused the disease were verified by Sanger sequencing. Results from sequencing revealed mutations in the *SLC37A4* and *LPL* genes. In the *SLC37A4* gene, a compound heterozygous variation was observed: her mother carried a c.1043T>C (p.L348P) mutation in exon 10 (chromosomal location: chr11:118895981; transcript: NM_001164277) (Figure 1), and her father carried a c.572C>T (p.P191L) mutation in exon 6 (chromosomal location: chr11:118898391; transcript: NM_001164277) (Figure 2). In addition, there was a novel c.483delA (p.A162Pfs^*^10) frameshift mutation in exon 4 of the patient's *LPL* gene (chromosomal location: chr8:19810873-19810874; transcript: NM_000237), which came from her father's heterozygous carrier (Figure 3). Her father had mild-to-moderate TG concentrations (355 mg/dl). These results were confirmed by Sanger sequencing.

Pathogenicity prediction analysis was performed using the Sorting Intolerant From Tolerant (SIFT; http://provean.jcvi.org/protein_batch_submit.php?species¼human), which sorts intolerant from tolerant amino acid substitutions, and PolyPhen-2 (http://genetics.bwh.harvard.edu/pph2/) platforms. The SIFT and PolyPhen-2 prediction programs indicated that these mutations were likely harmful.

The patient was switched from breastfeeding to lactose-free and MCT-enriched formula milk, followed by a subcutaneous injection of granulocyte colony-stimulating factor (G-CSF). In follow-up appointments, there was a dramatic decrease in hypertriglyceridemia within 5 days, as well as an increase in the neutrophil count after MCT and G-CSF therapy.

## Discussion

In this study, we reported a case of an infant who presented with typical features of GSD Ib, along with neutropenia, recurrent infections, and severe hypertriglyceridemia. Genomic sequencing revealed that the infant carried a novel c.483delA (p. A162Pfs^*^10) frameshift mutation in exon 4 of her *LPL* gene and compound heterozygous mutations [a c.1043T>C (p.L348P) mutation and a c.572C>T (p.P191L) mutation] in the *SLC37A4* gene. MCT milk application successfully treated her severe hypertriglyceridemia.

Previous studies have identified a c.572C>T (p.P191L) mutation in the *SLC37A4* gene. In a Chinese family with GSD Ib, the *G6PT1* gene was analyzed, and the results indicated that the family members carried maternal c.572C>T (p.P191L) mutation and paternal c.446G>A (p.G149E) mutation. Recently, a Chinese patient with GSD Ib was found compoundly heterozygous for the c.572C>T (p.P191L) (maternal source) mutation and a novel paternal c.359C>T (p.P120L) mutation (9). However, the heterozygous mutation of c.1043T>C (p.L348P) from the paternal source has not been reported before. In this study, the patient carried a c.1043T>C (p.L348P) mutation (maternal source) and a c.572C>T (p.P191L) mutation (paternal source).

Hypertriglyceridemia is defined by fasting serum TG levels of 150 mg/dl or more, which is a common lipid abnormality in clinical practice and is correlated with an elevated risk of cardiovascular disease (12). Single-nucleotide variants and large-scale copy number variants can both result in mild to moderate or severe hypertriglyceridemia, and different monogenic variants resulting in severe hypertriglyceridemia have been reported (7). *LPL* deficiency is a rare disorder caused by a loss of function of the *LPL* gene, and patients with *LPL* deficiency usually have marked hypertriglyceridemia. It was demonstrated that homozygous missense mutations (L252V) were found in the *LPL* gene of a newborn with hypertriglyceridemia in China (13). A recent study by Qin et al. (14) supported that a mutation of the *LPL* gene (c.836T>G) (p.Leu279Arg) contributed to severe hypertriglyceridemia in a Chinese infant. In addition, L279V mutation in the *LPL* gene and a reported mutation (A98T) compound heterozygote were proven to inhibit *LPL* enzymatic activity and lead to severe hypertriglyceridemia and acute pancreatitis (15). In the present study, a novel c.483delA (p.A162Pfs^*^10) frameshift mutation was found in *LPL* gene exon 4 of the infant with severe hypertriglyceridemia. The variant was novel but caused a stop in the reading frame. This was likely to be very pathogenic even though the LPL activity was not tested. Pedigree analysis suggested that the father had heterozygous variation in this locus, while the mother had no variation in this locus. In addition, the father with the same heterozygous mutation has mild to moderate TG concentrations. These findings suggested that the mutation in the *LPL* gene in GSD Ib patients might be directly correlated with the severe hypertriglyceridemia phenotype.

The efficacy of MCT milk for patients with GSD Ib has always been controversial (11). It was reported that patients with GSD Ib had hypertriglyceridemia that is often resistant to MCT (16). Das et al. (17) investigated the effects of an MCT-enriched diet on metabolic control or growth in patients with GSD 1 and suggested that MCT supplementation decreased TG levels in young cases and had a beneficial effect on the metabolic management of GSD 1 patients. In the current study, MCT milk application successfully treated severe hypertriglyceridemia of the patient.

## Conclusions

In conclusion, we found a novel frameshift mutation in the *LPL* gene that led to severe hypertriglyceridemia in a Chinese infant with GSD Ib, who was successfully treated with MCT milk.

## Data Availability Statement

The original contributions presented in the study are included in the article/supplementary material, further inquiries can be directed to the corresponding authors.

## Ethics Statement

The studies involving human participants were reviewed and approved by this study was approved by the Ethics Committee of Zibo Central Hospital, Shandong First Medical University. Written informed consent to participate in this study was provided by the participants' legal guardian/next of kin.

## Author Contributions

JZ and FengyW: conception and design of the research. FenglW, XZ, and YY: acquisition of data, analysis and interpretation of data, and statistical analysis. FengyW: drafting the manuscript. JZ: revision of manuscript for important intellectual content. All of the authors read and approved the final manuscript.

## Conflict of Interest

The authors declare that the research was conducted in the absence of any commercial or financial relationships that could be construed as a potential conflict of interest.

## Publisher's Note

All claims expressed in this article are solely those of the authors and do not necessarily represent those of their affiliated organizations, or those of the publisher, the editors and the reviewers. Any product that may be evaluated in this article, or claim that may be made by its manufacturer, is not guaranteed or endorsed by the publisher.

## Supplementary Material

Supplementary Figure 1 Results for liver color ultrasound.

## References

[1] Wicker C Roda C Perry A Arnoux JB Brassier A Castelle M. Infectious and digestive complications in glycogen storage disease type Ib: Study of a French cohort. Mol Genet Metab Rep. (2020) 23:100581. 10.1016/j.ymgmr.2020.100581 32300528 PMC7152669

[2] Sim SW Weinstein DA Lee YM Jun HS. Glycogen storage disease type Ib: role of glucose-6-phosphate transporter in cell metabolism and function. FEBS Lett. (2020) 594:3–18. 10.1002/1873-3468.13666 31705665

[3] Chen SY Pan CJ Nandigama K Mansfield BC Ambudkar SV Chou JY. The glucose-6-phosphate transporter is a phosphate-linked antiporter deficient in glycogen storage disease type Ib and Ic. FASEB J. (2008) 22:2206–13. 10.1096/fj.07-104851 18337460

[4] Chou JY Cho JH Kim GY Mansfield BC. Molecular biology and gene therapy for glycogen storage disease type Ib. J Inherit Metab Dis. (2018) 41:1007–14. 10.1007/s10545-018-0180-5 29663270

[5] Dron JS Wang J Cao H McIntyre AD Iacocca MA Menard JR. Severe hypertriglyceridemia is primarily polygenic. J Clin Lipidol. (2019) 13:80–8. 10.1016/j.jacl.2018.10.006 30466821

[6] Yamamoto T Gotoda T. Polygenic architecture of common severe Hypertriglyceridemia. J Atheroscler Thromb. (2020) 27:1255–56. 10.5551/jat.ED133 32493883 PMC7840165

[7] Dron JS Hegele RA. Genetics of hypertriglyceridemia. Front Endocrinol. (2020) 11:455. 10.3389/fendo.2020.00455 32793115 PMC7393009

[8] Rodrigues R Artieda M Tejedor D Martínez A Konstantinova P Petry H. Pathogenic classification of LPL gene variants reported to be associated with LPL deficiency. J Clin Lipidol. (2016) 10:394–409. 10.1016/j.jacl.2015.12.015 27055971

[9] Zhang Y Sun H Wan N. Mutation analysis of SLC37A4 in a patient with glycogen storage disease-type Ib. J Int Med Res. (2019) 47:5996–6003. 10.1177/0300060519867819 31617422 PMC7045669

[10] Hauenschild A Bretzel RG Schnellkretschmer H Kloer HU Hardt PD Ewald N. Successful treatment of severe Hypertriglyceridemia with a formula diet rich in omega-3 fatty acids and medium-chain triglycerides. Ann Nutr Metab. (2010) 56:170–5. 10.1159/000283561 20150726

[11] Nagasaka H Hirano K Ohtake A Miida T Takatani T Murayama K. Improvements of hypertriglyceridemia and hyperlacticemia in Japanese children with glycogen storage disease type Ia by medium-chain triglyceride milk. Eur J Pediatr. (2007) 166:1009–16. 10.1007/s00431-006-0372-0 17206455

[12] Oh RC Trivette ET Westerfield KL. Management of hypertriglyceridemia: common questions and answers. Am Fam Physician. (2020) 102:347–54. 32931217

[13] Chan AO But W Lau GT Tse WY Shek CC. A novel nonsense mutation in the LPL gene in a Chinese neonate with hypertriglyceridemia. Clin Chim Acta. (2006) 368:120–4. 10.1016/j.cca.2005.12.020 16460718

[14] Qin Y Wei A Shan Q Xian X Wu Y Liao L. Rare LPL gene missense mutation in an infant with hypertriglyceridemia. J Clin Lab Anal. (2018) 32:e22414. 10.1002/jcla.22414 29479812 PMC6817128

[15] Chen T Xie S Jin R Huang ZM. A novel lipoprotein lipase gene missense mutation in Chinese patients with severe hypertriglyceridemia and pancreatitis. Lipids Health Dis. (2014) 13:52–2. 10.1186/1476-511X-13-52 24646025 PMC3983885

[16] Derks T van Rijn M. Lipids in hepatic glycogen storage diseases: pathophysiology, monitoring of dietary management and future directions. J Inherit Metab Dis. (2015) 38:537–43. 10.1007/s10545-015-9811-2 25633903 PMC4432100

[17] Das A Lücke T Meyer U Hartmann H Illsinger S. Glycogen storage disease type 1: impact of medium-chain triglycerides on metabolic control and growth. Ann Nutr Metab. 56:225–32. 10.1159/000283242 20357432

