# Supplementary figures and images for: A Novel Lipoprotein Lipase Mutation in an Infant With Glycogen Storage Disease Type-Ib and Severe Hypertriglyceridemia
Source: Front Pediatr. 2021 Aug 17;9:671536. doi: 10.3389/fped.2021.671536 (PMC8416156; doi:10.3389/fped.2021.671536)

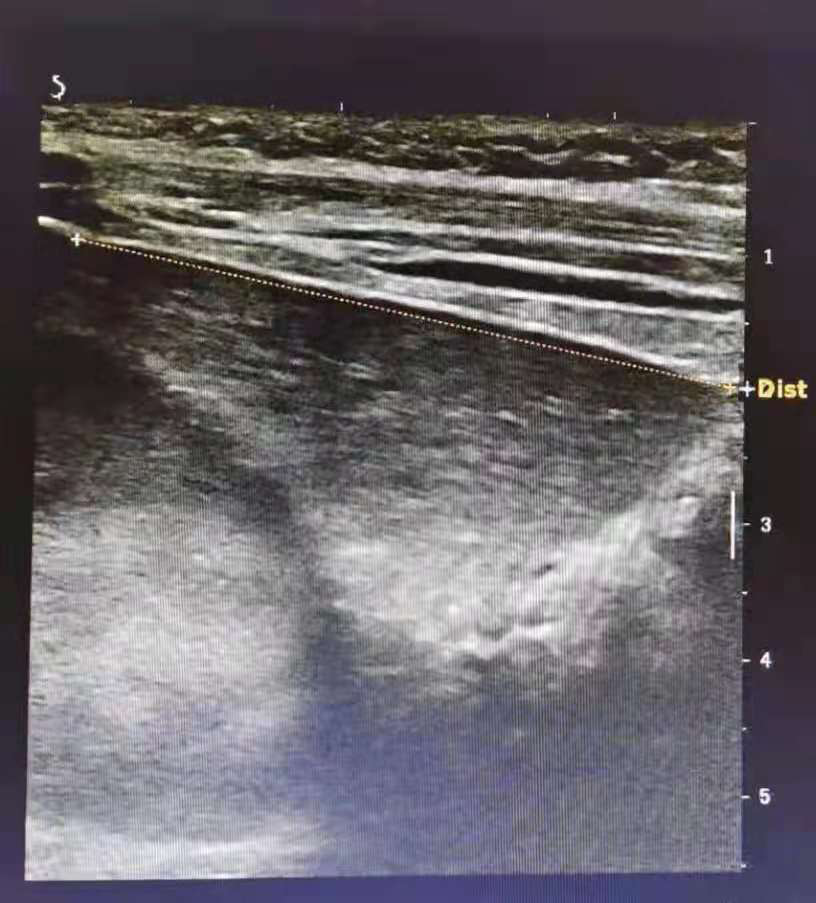

Supplement: Supplementary Figure 1 — Results for liver color ultrasound. [file Image_1.tif]
